# Self-reported sleep characteristics associated with dementia among rural-dwelling Chinese older adults: a population-based study

**DOI:** 10.1186/s12883-021-02521-0

**Published:** 2022-01-03

**Authors:** Rui Liu, Shi Tang, Yongxiang Wang, Yi Dong, Tingting Hou, Yifei Ren, Lin Cong, Keke Liu, Yu Qin, Shireen Sindi, Yifeng Du, Chengxuan Qiu

**Affiliations:** 1 Department of Neurology, Shandong Provincial Hospital, Cheeloo College of Medicine, Shandong University, No. 324 Jingwuweiqi Road, 250021 Jinan, Shandong People’s Republic of China; 2 Department of Neurology, Shandong Provincial Hospital Affiliated to Shandong First Medical University, Jinan, Shandong People’s Republic of China; 3 Shandong Provincial Clinical Research Center for Neurological Diseases, Jinan, Shandong People’s Republic of China; 4 Shandong Academy of Clinical Medicine, Shandong Provincial Hospital Affiliated to Shandong First Medical University, Jinan, Shandong People’s Republic of China; 5 Aging Research Center and Center for Alzheimer Research, Department of Neurobiology, Care Sciences and Society, Karolinska Institutet-Stockholm University, Stockholm, Sweden; 6 Division of Clinical Geriatrics, Department of Neurobiology, Care Sciences and Society, Karolinska Institutet, Stockholm, Sweden; 7 Neuroepidemiology and Ageing Research Unit, School of Public Health, Imperial College London, London, UK

**Keywords:** Older adults, Sleep, Cognitive function, Dementia, Alzheimer’s disease, Population-based study

## Abstract

**Background:**

Sleep characteristics associated with dementia are poorly defined and whether their associations vary by demographics and *APOE* genotype among older adults are unclear.

**Methods:**

This population-based cross-sectional study included 4742 participants (age ≥ 65 years, 57.1% women) living in rural China. Sleep parameters were measured using the self-rated questionnaires of the Pittsburgh Sleep Quality Index and Epworth Sleepiness Scale. Global cognitive function was assessed with the Mini-Mental State Examination (MMSE). Dementia was diagnosed following the Diagnostic and Statistical Manual of Mental Disorders, Fourth Edition, criteria, and the National Institute on Aging-Alzheimer’s Association criteria for Alzheimer’s disease (AD). Data were analysed using multiple logistic and general linear regression models.

**Results:**

Dementia was diagnosed in 173 participants (115 with AD). Multivariable-adjusted odds ratio (OR) of dementia was 1.71 (95%CI, 1.07-2.72) for sleep duration ≤4 h/night (vs. > 6-8 h/night), 0.76 (0.49-1.18) for > 4-6 h/night, 1.63 (1.05-2.55) for > 8 h/night, 1.11 (1.03-1.20) for lower sleep efficiency (per 10% decrease), and 1.85 (1.19-2.89) for excessive daytime sleepiness. Very short sleep duration (≤4 h/night), lower sleep efficiency, and excessive daytime sleepiness were significantly associated with being diagnosed with AD (multivariable-adjusted OR range = 1.12-2.07; *p* < 0.05). The associations of sleep problems with dementia and AD were evident mainly among young-old adults (65-74 years) or *APOE* ε4 carriers. Among dementia-free participants, these sleep characteristics were significantly associated with a lower MMSE score.

**Conclusions:**

Self-reported sleep problems in dementia are characterized by very short or long sleep duration, low sleep efficiency, and excessive daytime sleepiness, especially among young-old people and *APOE* ε4 carriers.

**Trial registration:**

ChiCTR1800017758 (Aug 13, 2018).

**Supplementary Information:**

The online version contains supplementary material available at 10.1186/s12883-021-02521-0.

## Background

Since the 1950s, China has experienced faster population aging than most countries in the world [1]. According to the Seventh National Population Census (2020), older people (age ≥ 65 years) accounted for 13.5% of China’s total population [2]. Along with rapid population aging, the age-related disorders such as cognitive impairment and dementia become increasingly common. For instance, a nationwide survey suggested that mild cognitive impairment and dementia affected 20.80 and 5.14%, respectively, of Chinese older adults [3]. In addition, as people age, sleep patterns change as well. The age-related changes in sleep patterns are characterized by a shorter sleep duration, longer sleep latency, lower sleep efficiency, worse sleep quality, and being more prone to daytime sleepiness compared with young adults [4]. Population-based studies showed that 57.8% of older adults reported poor sleep quality [5], and 13.2% reported excessive daytime sleepiness (EDS) [6]. Sleep problems and dementia both are increasingly common as people age, posing a tremendous burden to the families and the society at large [3, 7].

The associations between sleep characteristics and cognitive function have attracted great attention in recent years. Some longitudinal studies have shown that sleep problems (e.g., short or long sleep duration, prolonged sleep latency, and EDS), which are common among patients with dementia living in care homes [8], are also risk factors for dementia [9, 10]. The associations between poor sleep and dementia were supported in a meta-analysis and systematic review [11]. These findings indicate a potential bidirectional association between poor sleep quality and dementia. Several studies showed that some sleep characteristics such as extreme sleep duration, prolonged sleep latency, low sleep efficiency, and EDS were associated with poor cognitive function [12–14]. However, their independent associations remain inconsistent. For example, some population-based studies suggested an association of long, but not short, sleep duration with worse global cognitive performance in older adults [15, 16]. However, the population-based study of the Outcomes of Sleep Disorders in Older Men (MrOS Sleep Study) suggested that both short and long self-reported sleep durations were associated with worse global cognitive function [17]. In addition, most of the previous population-based cross-sectional studies have focused on the associations between sleep characteristics and cognitive performance rather than dementia [18, 19]. Furthermore, the majority of the previous studies have investigated the sleep characteristics of patients with dementia from clinical settings or care homes [8, 20]. Few population-based cross-sectional studies have assessed the associations of sleep characteristics with dementia and Alzheimer’s disease (AD) among older adults in China, especially those living in the rural communities with limited education and low socioeconomic status. Studying sleep features associated with dementia in rural residents is important because individuals from rural areas were more susceptible to dementia and AD [3], and more likely to have poor sleep, compared to older adults living in urban areas [21].

In addition, population-based studies have shown that poor sleep quality and dementia are more common in women than in men [3, 5], and in people with low than high education [3, 22]. Thus, the associations between sleep characteristics and dementia may be related to demographic factors (e.g., sex and education). In addition, apolipoprotein E (*APOE*) ε4 allele, as a well-established genetic risk factor for dementia [23], has been associated with a short sleep duration [24]. Taken together, whether the association of sleep characteristics with dementia varies by demographics (age, sex, and education) and *APOE* genotype remains unclear. This is important because clarifying this issue may help better characterize the abnormal sleep patterns associated with cognitive disorders in older adults.

Therefore, in this population-based cross-sectional study of Chinese older adults, we sought to (1) characterize the sleep features associated with dementia and poor global cognitive performance and (2) further explore the interactions of sleep characteristics with demographic factors and *APOE* genotype on cognitive outcomes. We hypothesize that short and long sleep duration, poor sleep quality, low sleep efficiency, prolonged sleep latency, and EDS are associated with dementia in older adults, and that the associations may vary depending on demographic factors and *APOE* ε4 status.

## Methods

### Study design and participants

This population-based cross-sectional study included participants in the baseline assessments of the Multimodal Interventions to Delay Dementia and Disability in Rural China (MIND-China) [25, 26], a participating project in the World-Wide FINGERS Network [27]. MIND-China was conducted by Shandong Provincial Hospital affiliated to Shandong University Cheeloo College of Medicine, in collaboration with local Yanlou Town Hospital in Yanggu County, Shandong.

Eligible participants included all registered residents (*n* = 7698) who were aged ≥60 years by the end of 2017 and living in the 52 villages of Yanlou Town, Yanggu County, western Shandong Province. In March-September 2018, the baseline assessments for MIND-China were integrated with the annual health check-up program provided by local government for residents who reached 65 years of age. In addition, residents who were aged 60-64 years were specifically invited for the MIND-China study. Because we aimed to study late-onset dementia and the participating rate was relatively low, participants aged 60-64 years (*n* = 1170) were excluded from the analyses. Of the 6528 eligible residents who were aged ≥65 years, 1282 (19.6%) were excluded due to death prior to the examination (*n* = 123), refusal (*n* = 829), severe mental illnesses (*n* = 23), and not reachable (*n* = 307). Thus, a total number of 5246 participants (80.4% of all the eligible residents who were aged ≥65 years) were examined for MIND-China.

**Fig. 1:**
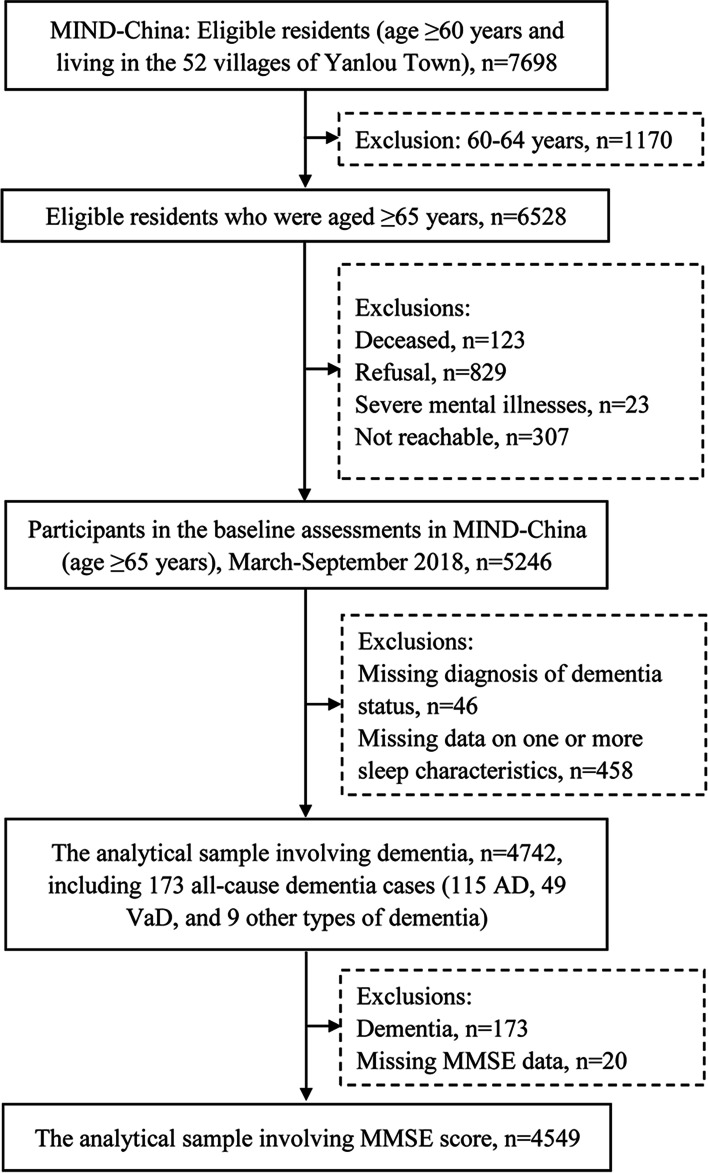
Flowchart of the study participants. *Abbreviations: AD* Alzheimer’s disease, *MIND-China* Multimodal Interventions to Delay Dementia and Disability in Rural China, *MMSE* Mini-Mental State Examination, *VaD* vascular dementia

Of the 5246 participants who were aged ≥65 years, 504 were excluded due to missing diagnosis of dementia status (*n* = 46) or missing data on one or more sleep characteristics (*n* = 458, including 129 with dementia), leaving 4742 (72.6% of all the 6528 eligible residents who were aged ≥65 years) for the analyses involving dementia. When the analyses involved global cognitive performance among participants who were free from dementia, we further excluded 173 participants who were diagnosed with dementia, and 20 participants who had missing data on the Mini-Mental State Examination (MMSE) score [28, 29], leaving 4549 participants for the analyses involving MMSE score (Fig. 1).

Compared to participants who were excluded from our analyses (*n* = 504), those included in the analytical sample (*n* = 4742) were younger (mean age, 71.27 vs. 76.18 years, *p* < 0.001) and more educated (mean years of formal schooling: 3.21 vs. 1.97, *p* < 0.001), but the two groups did not differ significantly by sex (women: 57.1% vs. 58.9%, *p* = 0.714).

MIND-China has been approved by the Ethics Committee of Shandong Provincial Hospital in Jinan, Shandong. Prior to the assessments, written informed consent was obtained from all participants, or informed consent was provided by proxy for persons when they were ‘deemed cognitively impaired’. Research within MIND-China has been conducted in accordance with the principles expressed in the Declaration of Helsinki. MIND-China was registered in the Chinese Clinical Trial Registry (registration no.: ChiCTR1800017758).

### Data collection

Data were collected by trained staff through face-to-face interviews, clinical examinations, neuropsychological tests, and laboratory tests, as previously reported [25, 26]. In brief, we collected data following a structured questionnaire that included information on demographic factors (e.g., age, sex, and education), lifestyle factors (e.g., alcohol drinking, smoking, and leisure-time physical activity), health conditions (e.g., hypertension, diabetes, and coronary heart disease), use of medications (e.g., antihypertensive and hypoglycaemic agents), sleep characteristics, and cognitive function. All medications were classified according to the Anatomical Therapeutic Chemical (ATC) classification system, as previously reported [25]. Weight and height were measured in light clothes without shoes. Sitting arterial blood pressure was measured on the right arm using an electronic sphygmomanometer (HEM-7127 J, Omron Corporation, Kyoto, Japan) after at least a 5-min rest. The 12-lead resting electrocardiogram (ECG) was recorded by an electrocardiograph (CM300, COMEN, Shenzhen, China) and then analysed by a physician. Peripheral blood samples were taken after an overnight fast. Blood glucose, total cholesterol (TC), triglycerides (TG), low-density lipoprotein cholesterol (LDL-C), and high-density lipoprotein cholesterol (HDL-C) were measured using an Automatic Biochemical Analyzer (CS-600B, DIRUI Corporation, Changchun, China) at the Yanlou Town Hospital laboratory. *APOE* genotyping was performed using multiple-polymerase chain reaction amplification (iGeneTech Bioscience Co., Ltd., Beijing, China).

### Sleep characteristics

Sleep characteristics (e.g., sleep duration, sleep quality, sleep efficiency, and sleep latency) were assessed via in-person interviews (self-report) using the validated Chinese version of the Pittsburgh Sleep Quality Index (PSQI) [30]. The PSQI is a 19-item self-rated questionnaire to assess sleep quality during the last month, which includes seven domains, i.e., subjective sleep quality, sleep latency, sleep duration, habitual sleep efficiency, sleep disturbances, use of sleeping medication, and daytime dysfunction. The score for each domain ranges from 0 to 3, and the total PSQI score ranges from 0 to 21. A higher score indicates poorer sleep quality. Poor sleep quality is defined as a total PSQI score > 5. The Chinese version of PSQI showed good internal consistency (Cronbach’s α = 0.82-0.83) and test-retest reliability (r = 0.85) [31]. The PSQI has been recommended for assessing sleep quality of people with dementia [32].

The sleep duration (hours/night) was categorized as very short (≤4), short (> 4 to 6), normal (> 6 to 8, reference), and long (> 8) sleep duration, consistent with previous studies [33, 34]. Sleep efficiency is defined as the percentage of time spent on actual sleep while in bed. Sleep latency refers to the minutes taken to fall asleep at night, and prolonged sleep latency is defined as the latency > 30 min [13].

Daytime sleepiness was assessed via the in-person interviews (self-report) using the Chinese version of the Epworth Sleepiness Scale (ESS) [35]. ESS is an 8-item Likert scale to measure the daytime sleepiness of the respondent in eight daily situations. The score of each question ranges from 0 to 3, leading to the total ESS score ranging from 0 to 24. A higher score indicates greater daytime sleepiness. EDS is defined as the total ESS score > 10. The Chinese version of the ESS showed good internal consistency (Cronbach’s α = 0.81) and test-retest reliability (r = 0.74) [36]. The ESS was also recommended for evaluating EDS among people with dementia [32].

### Assessments of cognitive function, dementia, and dementia subtypes

A neuropsychological test battery, mainly including MMSE, the self-reported Ascertain Dementia 8-item Questionnaire, the Auditory Verbal Learning Test, the Category Verbal Fluency Test, the Forward and Backward Digit Span Test, and the Trail Making Test A and B, was used to assess subjective cognitive complaints, global cognitive function, and function of specific cognitive domains (e.g., memory, language, attention, and executive function). Global cognitive function was evaluated with the validated Chinese version of the MMSE [28]. MMSE score ranges from 0 to 30, with a higher score indicating better global cognitive performance.

Dementia was diagnosed according to the Diagnostic and Statistical Manual of Mental Disorders, Fourth Edition (DSM-IV), criteria [37], following a three-step diagnostic procedure [38]. In brief, trained clinicians and interviewers conducted routine clinical examination and comprehensive assessments for each participant following the standard procedure. The assessments included medical history, a neurocognitive assessment battery, and activities of daily living (ADLs). Then, the neurologists specialized in dementia diagnosis and care reviewed all the information collected from the previous assessments, and made a preliminary judgement of dementia for participants who were suspected to have dementia. Finally, the neurologists conducted additional face-to-face interviews with those participants who were suspected to have dementia, and reassessed their medical history, cognitive status, ADLs, and whenever available, neuroimaging data. If the participants were not able to undergo the interview due to severe cognitive impairment or were not available for the face-to-face interviews (about 13%), the neurologists interviewed their family members, neighbors, or village doctors (who provide primary care services to local residents). Following all the interviews and assessments, the neurologists made the diagnosis of dementia according to the DSM-IV criteria [37]. In case of uncertainty, a senior neurologist (L.C.) was consulted, and a consensus was made on whether the participant had dementia. Dementia was further classified into AD according to the National Institute on Aging-Alzheimer’s Association (NIA-AA) criteria for probable AD [39] and vascular dementia (VaD) following the National Institute of Neurological Disorders and Stroke and the Association Internationale pour la Recherche et l’Enseignement en Neurosciences (NINDS-AIREN) criteria for probable VaD [40]. Dementia cases who could not be classified as either AD or VaD were considered other types of dementia.

### Assessment of covariates

Body mass index (BMI) was calculated as weight in kilograms divided by the square of height in metres. BMI (kg/m^2^) was categorized as underweight (< 18.5), normal (18.5-23.9), overweight (24-27.9), and obese (≥28), following the criteria for the Chinese population [26]. Alcohol consumption and smoking status were categorized as current, former, and never drinking or smoking, respectively. Leisure-time physical activity was defined as doing any type of physical activity during leisure time at least once a week. Hypertension was defined as arterial blood pressure ≥ 140/90 mmHg or current use of antihypertensive agents (ATC codes C02, C03, and C07-C09). Diabetes was defined as the fasting blood glucose ≥7.0 mmol/L, or taking antidiabetic agents (ATC code A10), or a self-reported history of diabetes. Dyslipidemia was defined as TC ≥6.22 mmol/L, or TG ≥2.27 mmol/L, or LDL-C ≥ 4.14 mmol/L, or HDL-C < 1.04 mmol/L, or use of hypolipidemic agents (ATC code C10) [26]. Coronary heart disease was defined according to self-reported history or ECG examination, including angina, myocardial infarction, coronary angioplasty, and coronary artery bypass grafting. Stroke was defined according to self-reported history and neurological examination. Depressive symptoms were assessed using the 15-item Geriatric Depression Scale (GDS-15) [41]. The presence of depressive symptoms was defined as a GDS-15 score ≥ 5. Hypnotics use was defined as the use of hypnotics and sedatives (ATC code N05C). *APOE* genotype was dichotomized as carriers vs. non-carriers of the *APOE* ε4 allele.

### Statistical analysis

Characteristics of study participants by dementia status were compared using Mann-Whitney *U* test for continuous variables, and chi-square test for categorical variables. Binary logistic regression models were used to examine the associations of self-reported sleep characteristics with all-cause dementia and AD. We did not analyse VaD (*n* = 49) and other types of dementia (*n* = 9) separately owing to relatively few cases. General linear models were used to analyse the associations of self-reported sleep characteristics with MMSE score among dementia-free participants. We reported the main results from two models: Model 1 was adjusted for age, sex, and education, and Model 2 was additionally adjusted for BMI, alcohol consumption status, smoking status, leisure-time physical activity, hypertension, diabetes, dyslipidemia, coronary heart disease, stroke, depressive symptoms, hypnotics use, and *APOE* genotype. Missing values of each of covariates were treated as a dummy variable in order to maximize the sample size. Results for the category of missing values did not provide additional insight.

We then tested the statistical interactions of self-reported sleep characteristics with age groups (< 75 vs. ≥75 years), sex, education (illiteracy vs. non-illiteracy), or *APOE* ε4 allele (carriers vs. non-carriers) on all-cause dementia and AD by simultaneously entering the independent variables and their cross-product term into the same model. We performed stratified analyses by demographics and *APOE* genotype.

IBM SPSS Statistics for Windows, Version 22.0 (IBM Corp., Armonk, NY) was used for all analyses. Two-tailed *p* < 0.05 was considered to be statistically significant.

## Results

### Characteristics of study participants

Of the 4742 participants, 173 (3.6%) were diagnosed with dementia, including 115 (2.4%) with AD, 49 (1.0%) with VaD, and 9 (0.2%) with other types of dementia. The mean age of all participants was 71.27 years (standard deviation 4.99), 57.1% were women, and 38.7% were illiterate. Compared with dementia-free participants, participants with dementia were older, less educated, more likely to be women and underweight, less likely to be obese, smoke, and drink alcohol, had a higher prevalence of diabetes, dyslipidemia, coronary heart disease, stroke, and depressive symptoms, more likely to use hypnotics, had a higher PSQI score and a higher ESS score, and a lower MMSE score (*p* < 0.05). The two groups had no significant differences in leisure-time physical activity, hypertension, *APOE* ε4 status, and sleep duration (*p* > 0.05) (Table 1).

**Table 1:** Characteristics of study participants by dementia status

Characteristics	Total sample	Dementia status		
	(*n* = 4742)	No (*n* = 4569)	Yes (*n* = 173)	*p*^a^
Age (years)	71.27 (4.99)	71.14 (4.87)	74.63 (6.69)	< 0.001
Women, n (%)	2707 (57.1)	2581 (56.5)	126 (72.8)	< 0.001
Education (years)	3.21 (3.44)	3.28 (3.45)	1.45 (2.55)	< 0.001
BMI (kg/m^2^), n (%)^b^				0.005
< 18.5	150 (3.2)	138 (3.0)	12 (7.0)	
18.5-23.9	1814 (38.4)	1743 (38.3)	61 (35.7)	
24-27.9	1817 (38.5)	1753 (38.5)	74 (43.3)	
≥ 28.0	938 (19.9)	914 (20.1)	24 (14.0)	
Alcohol drinking, n (%)				< 0.001
Never	2894 (61.0)	2759 (60.4)	135 (78.0)	
Former	452 (9.5)	437 (9.6)	15 (8.7)	
Current	1396 (29.4)	1373 (30.1)	23 (13.3)	
Smoking, n (%)				0.001
Never	3040 (64.1)	2908 (63.6)	132 (76.3)	
Former	988 (20.8)	969 (21.2)	19 (11.0)	
Current	714 (15.1)	692 (15.1)	22 (12.7)	
Leisure-time physical activity, n (%)	3174 (66.9)	3061 (67.0)	113 (65.3)	0.645
Hypertension, n (%)^b^	3154 (67.0)	3038 (67.0)	116 (67.4)	0.911
Diabetes, n (%)	684 (14.4)	643 (14.1)	41 (23.7)	< 0.001
Dyslipidemia, n (%)	1118 (23.6)	1063 (23.3)	55 (31.8)	0.010
Coronary heart disease, n (%)	1032 (21.8)	981 (21.5)	51 (29.5)	0.012
Stroke, n (%)	747 (15.8)	687 (15.0)	60 (34.7)	< 0.001
Depressive symptoms, n (%)^b^	459 (9.8)	401 (8.9)	58 (37.4)	< 0.001
Hypnotics use, n (%)	233 (4.9)	215 (4.7)	18 (10.4)	0.001
*APOE* ε4 carriers, n (%)^b^	729 (15.9)	697 (15.7)	32 (18.6)	0.314
Sleep duration (h)	6.63 (1.71)	6.63 (1.67)	6.63 (2.44)	0.817
PSQI score	5.97 (4.18)	5.90 (4.12)	8.08 (5.08)	< 0.001
ESS score	4.44 (4.25)	4.38 (4.16)	6.12 (5.95)	0.002
MMSE score^b^	20.88 (6.03)	21.25 (5.74)	10.38 (4.27)	< 0.001

Data were mean (standard deviation), unless otherwise specified

^a^*p* value was for the test of differences between participants without and with dementia

^b^Numbers of subjects with missing values were 23 for BMI, 38 for hypertension, 72 for depressive symptoms, 143 for *APOE* genotype, and 33 for MMSE score. In subsequent analyses, a dummy variable was created for participants with missing data in each of these covariates

*Abbreviations: APOE* Apolipoprotein E gene, *BMI* Body mass index, *ESS* Epworth Sleepiness Scale, *MMSE* Mini-Mental State Examination, *PSQI* Pittsburgh Sleep Quality Index

### Associations of self-reported sleep characteristics with all-cause dementia and AD

Controlling for age, sex, and education, very short or long sleep duration, poor sleep quality, lower sleep efficiency, and EDS were significantly associated with an increased likelihood of all-cause dementia (Table 2, Model 1). When controlling for additional potential confounding factors, the associations with dementia remained statistically significant for all sleep characteristics, except poor sleep quality and prolonged sleep latency (Table 2, Model 2).

**Table 2:** Associations of self-reported sleep characteristics with all-cause dementia and Alzheimer’s disease (n = 4742)

Self-reported sleep characteristics	No. of subjects	All-cause dementia (n = 173)			Alzheimer’s disease (*n* = 115)		
		No. of cases	Odds ratio (95% confidence interval)		No. of cases	Odds ratio (95% confidence interval)	
			Model 1^a^	Model 2^b^		Model 1^a^	Model 2^b^
Sleep duration							
≤ 4 h	486	37	2.20 (1.42-3.41)^***^	1.71 (1.07-2.72)^*^	27	2.16 (1.29-3.62)^**^	1.78 (1.03-3.05)^*^
> 4 to 6 h	1371	37	0.94 (0.62-1.43)	0.76 (0.49-1.18)	26	0.96 (0.58-1.60)	0.83 (0.49-1.40)
> 6 to 8 h	2228	59	1.00 (Reference)	1.00 (Reference)	40	1.00 (Reference)	1.00 (Reference)
> 8 h	657	40	1.98 (1.30-3.02)^**^	1.63 (1.05-2.55)^*^	22	1.57 (0.91-2.68)	1.39 (0.80-2.44)
Sleep quality							
Good	2523	66	1.00 (Reference)	1.00 (Reference)	41	1.00 (Reference)	1.00 (Reference)
Poor	2219	107	1.59 (1.16-2.19)^**^	1.17 (0.82-1.67)	74	1.68 (1.13-2.50)^*^	1.27 (0.83-1.95)
Sleep efficiency (per 10% decrease)	4742	173	1.18 (1.09-1.27)^***^	1.11 (1.03-1.20)^**^	115	1.19 (1.09-1.30)^***^	1.12 (1.02-1.24)^*^
Sleep latency							
≤ 30 min	3434	103	1.00 (Reference)	1.00 (Reference)	62	1.00 (Reference)	1.00 (Reference)
> 30 min	1308	70	1.39 (1.01-1.92)^*^	1.15 (0.81-1.62)	53	1.65 (1.12-2.43)^*^	1.38 (0.91-2.08)
EDS							
No	4310	140	1.00 (Reference)	1.00 (Reference)	94	1.00 (Reference)	1.00 (Reference)
Yes	432	33	2.93 (1.95-4.42)^***^	1.85 (1.19-2.89)^**^	21	2.87 (1.73-4.75)^***^	2.07 (1.21-3.54)^**^

^a^Model 1 was adjusted for age, sex, and education

^b^Model 2 was additionally adjusted for body mass index, alcohol consumption status, smoking status, leisure-time physical activity, hypertension, diabetes, dyslipidemia, coronary heart disease, stroke, depressive symptoms, hypnotics use, and *APOE* genotype

^*^p < 0.05, ^**^p < 0.01, ^***^p < 0.001

*Abbreviations: EDS* Excessive daytime sleepiness

Similarly, very short sleep duration, poor sleep quality, lower sleep efficiency, prolonged sleep latency, and EDS were significantly associated with an increased likelihood of AD after controlling for age, sex, and education (Table 2, Model 1). In the fully-adjusted models, the associations between poor sleep quality and prolonged sleep latency with AD became non-significant (Table 2, Model 2). There was no significant association between long sleep duration and AD. Additionally adjusting for EDS did not alter the associations of nighttime sleep characteristics with all-cause dementia and AD (data not shown).

### Associations between self-reported sleep characteristics and global cognitive function in dementia-free participants

General linear regression analysis suggested that controlling for age, sex, and education, very short or long sleep duration, poor sleep quality, lower sleep efficiency, prolonged sleep latency, and EDS were significantly associated with a lower MMSE score among participants who were free of dementia (Table 3, Model 1). In the fully-adjusted models, the associations remained statistically significant for very short and long sleep duration, lower sleep efficiency, and EDS, but not for poor sleep quality and prolonged sleep latency (Table 3, Model 2). When additionally adjusting for EDS, the associations between nighttime sleep characteristics and global cognitive function were similar to those in Model 2 (data not shown).

**Table 3:** Associations between self-reported sleep characteristics and the Mini-Mental State Examination score among dementia-free participants (*n* = 4549)

Self-reported sleep characteristics	No. of subjects	β coefficient (95% confidence interval)	
		Model 1^a^	Model 2^b^
Sleep duration			
≤ 4 h	447	−0.97 (−1.40 - -0.53)^***^	− 0.86 (− 1.29 - -0.42)^***^
> 4 to 6 h	1326	− 0.13 (− 0.42 - 0.15)	− 0.09 (− 0.38 - 0.20)
> 6 to 8 h	2164	0.00 (Reference)	0.00 (Reference)
> 8 h	612	− 0.67 (− 1.04 - -0.29)^***^	− 0.61 (− 0.99 - -0.24)^**^
Sleep quality			
Good	2446	0.00 (Reference)	0.00 (Reference)
Poor	2103	−0.37 (− 0.61 - -0.12)^**^	−0.24 (− 0.49 - 0.02)
Sleep efficiency (per 10% decrease)	4549	−0.16 (− 0.23 - -0.09)^***^	−0.13 (− 0.20 - -0.06)^***^
Sleep latency			
≤ 30 min	3314	0.00 (Reference)	0.00 (Reference)
> 30 min	1235	−0.35 (− 0.64 - -0.07)^*^	−0.23 (− 0.51 - 0.06)
EDS			
No	4151	0.00 (Reference)	0.00 (Reference)
Yes	398	−1.28 (−1.71 - -0.84)^***^	− 1.11 (− 1.55 - -0.68)^***^

^a^Model 1 was adjusted for age, sex, and education

^b^Model 2 was additionally adjusted for body mass index, alcohol consumption status, smoking status, leisure-time physical activity, hypertension, diabetes, dyslipidemia, coronary heart disease, stroke, depressive symptoms, hypnotics use, and *APOE* genotype

^*^p < 0.05, ^**^p < 0.01, ^***^p < 0.001

*Abbreviations: EDS* Excessive daytime sleepiness

### Interactions of demographics and *APOE* genotype with self-reported sleep characteristics on dementia and AD

**Fig. 2:**
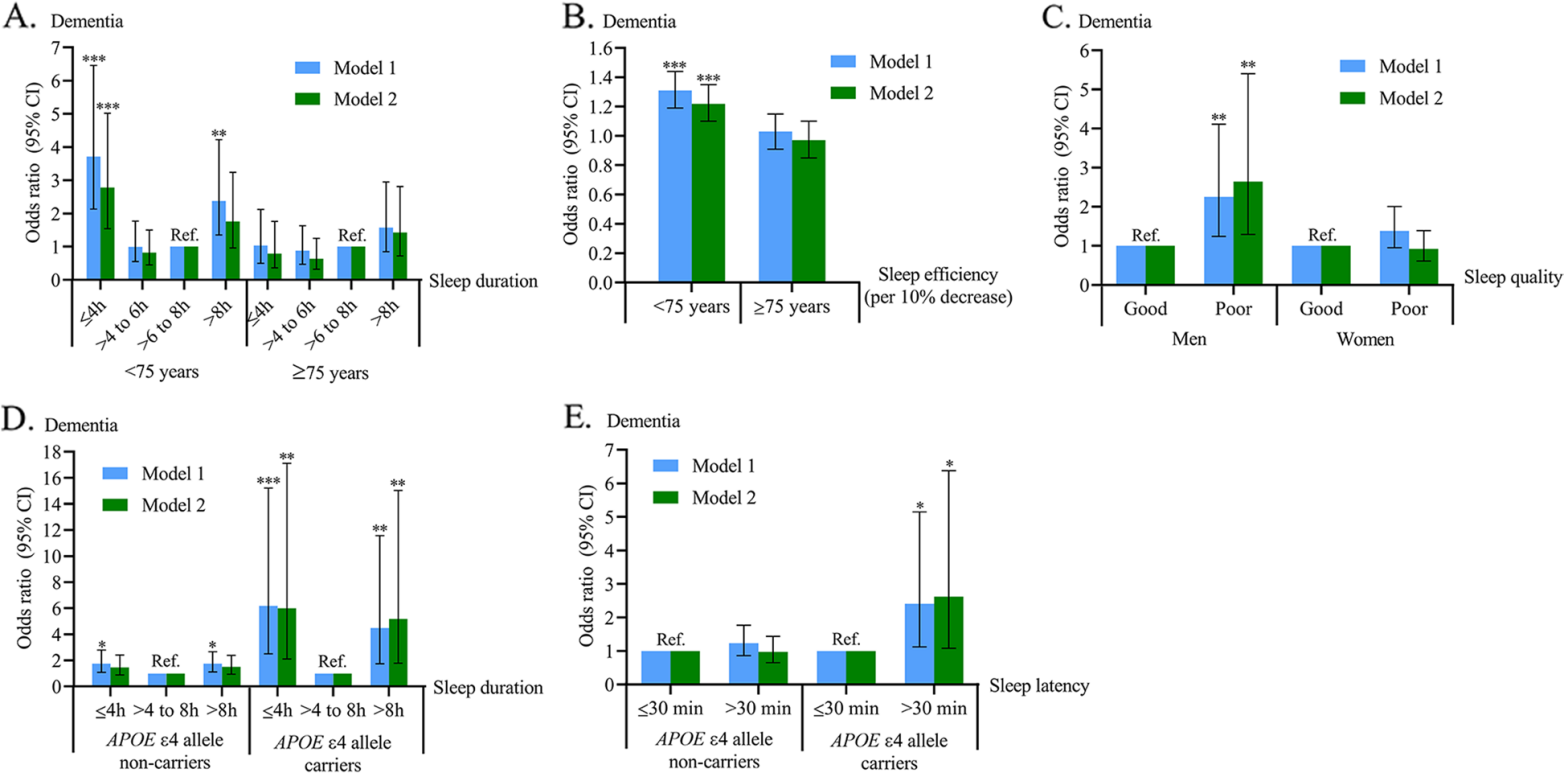
Associations between self-reported sleep characteristics and dementia by age groups, sex, and *APOE* ε4 status. **A** Sleep duration and dementia by age groups; **B** Sleep efficiency and dementia by age groups; **C** Sleep quality and dementia by sex; **D** Sleep duration and dementia by *APOE* ε4 status; **E** Sleep latency and dementia by *APOE* ε4 status. Model 1 was adjusted for age, sex, and education. Model 2 was additionally adjusted for body mass index, alcohol consumption status, smoking status, leisure-time physical activity, hypertension, diabetes, dyslipidemia, coronary heart disease, stroke, depressive symptoms, hypnotics use, and *APOE* genotype. ^*^p < 0.05, ^**^p < 0.01, ^***^p < 0.001. *Abbreviations: APOE* apolipoprotein E gene, *CI* confidence interval, *Ref.* reference

Figure 2 showed the results from stratified analyses, in which statistically significant interactions on the likelihood of dementia was detected. We reported the results from other interactive analyses and stratified analyses in the Additional file 1. These interaction and stratified analyses were summarized below.

We detected statistically significant interactions of age (< 75 vs. ≥75 years) with sleep duration (≤4 vs. > 6 to 8 h/night) and sleep efficiency (per 10% decrease) on the likelihood of dementia (*p* for interactions = 0.028 and 0.020, respectively). Further analyses stratified by age groups showed that, very short sleep duration and lower sleep efficiency were significantly associated with an increased likelihood of dementia among participants aged < 75 years, but not among those aged ≥75 years (Fig. 2A and B).

We examined the interactions of other sleep parameters with demographics and *APOE* ε4 allele on the likelihood of dementia. (1) We did not detect a statistical interaction of EDS with age groups on dementia (Supplementary Table 1, Additional file 1), but there was a statistical interaction between sex and sleep quality on dementia (*p* for interaction = 0.036), such that long sleep duration and EDS were associated with dementia only in women, while poor sleep quality was associated with dementia only in men (Fig. 2C and Supplementary Table 2, Additional file 1). (2) We did not detect statistical interactions between education and self-reported sleep characteristics on dementia. However, analyses stratified by education showed that the associations of lower sleep efficiency and EDS with dementia were significant only in illiterate persons, while the significant association of long sleep duration with dementia was found only in non-illiterate people (Supplementary Table 3, Additional file 1). (3) There were statistical interactions of *APOE* ε4 allele with sleep duration (≤4 vs. > 6 to 8 h/night) and sleep latency on the likelihood of dementia (*p* for interaction = 0.023 and 0.014, respectively). Analyses stratifying by *APOE* ε4 status suggested that extreme sleep duration, lower sleep efficiency, and prolonged sleep latency were significantly associated with dementia only in *APOE* ε4 allele carriers (Fig. 2D, E, and Supplementary Table 4, Additional file 1). Interactions and stratifying analyses for AD yielded results similar to those for all-cause dementia (Supplementary Tables 1-4, Additional file 1).

## Discussion

In this population-based study of older adults who were living in the rural communities in western Shandong Province, China, we found that self-reported very short or long sleep duration, lower sleep efficiency, and EDS were independently associated with a higher likelihood of all-cause dementia and AD. Furthermore, among dementia-free participants, these sleep characteristics were also associated with low global cognitive performance. In addition, the associations of sleep problems with dementia and AD were evident mainly among young-old adults (age 65-74 years) and *APOE* ε4 allele carriers. Taken together, self-reported sleep problems among people with dementia are characterized by very short or long sleep duration, lower sleep efficiency, and EDS, especially among young-old adults and *APOE* ε4 allele carriers.

To the best of our knowledge, this is the first cross-sectional population-based study in China to characterize self-reported sleep characteristics associated with all-cause dementia and AD that targets older adults living in the rural communities. Previous studies showed that the prevalence of dementia was higher in rural than urban populations in China, probably due to limited education and low socioeconomic status in rural residents [3]. Moreover, compared with urban elderly residents, rural-dwelling older adults were more likely to have poor sleep [21]. Older adults in the rural areas often engage in heavy farmland labour work, have limited access to health care, are more likely to suffer from somatic disorders, and have insufficient knowledge of sleep hygiene, which in turn may be linked to poor sleep quality and dementia [21, 42]. Given that very few population-based studies have targeted rural residents, the associations between sleep characteristics and dementia among rural-dwelling older adults deserve further investigation.

We found that self-reported very short or long sleep duration, lower sleep efficiency, and EDS were independently associated with dementia or AD. Indeed, the meta-analysis of population-based cohort studies showed that both long and short sleep duration were associated with an increased risk of dementia and AD, although the association with short sleep duration was less evident [43]. Few cross-sectional population-based studies have examined the independent associations between sleep characteristics and dementia. An Italian population-based study of older adults showed that EDS was associated with dementia [44], which is in line with our findings. By contrast, the population-based case-control study in Greece showed that self-reported night sleep duration was not associated with AD after adjusting for demographic factors, BMI, depression diagnosis, and benzodiazepines use [14]. Differences in study design, ethnicities, socioeconomic status, sleep-related questionnaires, and control of potential confounders might partly contribute to the inconsistent findings across studies.

In addition, we found that self-reported very short and long sleep duration, lower sleep efficiency, and EDS were associated with worse global cognitive function in dementia-free participants. The associations of very short and long self-reported sleep duration with worse global cognitive function are in a good agreement with results from the MrOS Sleep Study [17]. However, a cross-sectional study of Spanish older adults showed that long (≥11 h), but not short (≤6 h), sleep duration was associated with worse cognitive function [15]. However, this study did not separate night and daytime sleep, which was important considering that long naps were associated with worse cognitive function [45]. A population-based study of urban-dwelling Chinese older adults also found that longer, but not shorter, sleep duration was associated with worse cognitive function [16]. However, participants in that study had much longer average sleep duration (7.96 h) and higher mean MMSE score (26.1) than those in our study. Thus, differences in socioeconomic status, educational levels, and medical conditions between rural and urban populations may partly contribute to the different results. Our findings of the associations between lower sleep efficiency and EDS with worse global cognitive function were similar to the reports from previous studies [44, 46]. Besides the self-reported sleep characteristics, the association of objectively-measured low sleep efficiency with cognitive impairment had been reported in the Study of Osteoporotic Fractures [47], which is in line with our study. Notably, the associations of nighttime sleep characteristics with all-cause dementia, AD, and low global cognitive function were present independent of EDS, suggesting that their associations could not be explained by daytime sleep features (e.g., EDS).

Several potential mechanisms may explain the cross-sectional associations of self-reported sleep characteristics with dementia and worse cognitive function. Very short sleep duration is associated with brain amyloid-β (Aβ) deposition [48], increased tau protein in cerebrospinal fluid [49], and suppression of hippocampal neurogenesis [50]. Long sleep duration may reflect a proinflammatory state [51] and has been associated with more white matter hyperintensities [52], which may be the pathways linking to dementia. In addition, lower sleep efficiency and EDS are associated with Aβ deposition [53, 54]. On the other hand, Aβ deposition and tau phosphorylation aggregation in the hypothalamus may affect brain regions that regulate sleep, and thus, may cause sleep problems in patients with dementia [55].

Few studies have explored the potential interactions of sleep characteristics with demographic and genetic factors on dementia in older adults. We found that the associations of self-reported very short sleep duration and lower sleep efficiency with dementia and AD were evident in young-old (< 75 years), but not in old-old (≥75 years), individuals. The exact reasons for the age-varying associations are unclear. Previous studies have indeed shown that older adults with dementia and poor sleep quality may have a higher risk of mortality [56, 57], which might partly contribute to the lack of cross-sectional associations of sleep duration and sleep efficiency with dementia in the old-old group. We did not find a consistent pattern of the sleep parameters-dementia associations across sexes, which was similar to a previous study [58]. Data from the Framingham Heart Study observed an interaction between sleep duration and education (lower than high school vs. high school degree and above) on incident dementia, such that long sleep duration was associated with a higher risk of incident dementia only among participants without a high school degree [59]. We did not detect statistical interactions between education and self-reported sleep characteristics on dementia. However, our data did show that the associations of lower sleep efficiency and EDS with dementia were evident mainly in illiterate individuals, while the associations between long sleep duration and dementia were present only in non-illiterate people. Differences in the study design (e.g., cross-sectional vs. longitudinal study) and demographic features of the study participants (e.g., education) might partly contribute to the different findings. Furthermore, we found that very short and long sleep duration and prolonged sleep latency were associated with dementia and AD mainly among *APOE* ε4 allele carriers, which was in line with the potential that extreme sleep duration and carrying *APOE* ε4 allele may act additively or synergistically to be linked with dementia via common pathways such as Alzheimer pathology and neuroinflammation [60]. Previous studies have yielded mixed results with regard to whether the associations between sleep problems and dementia in older adults vary by *APOE* ε4 allele status. For instance, a cohort study showed that better sleep consolidation could attenuate the effect of *APOE* ε4 allele on AD risk, which is consistent with our cross-sectional study [61]. By contrast, another longitudinal study showed that the association between sleep problems and dementia existed only among non-carriers of the *APOE* ε4 allele [58]. Thus, the complex interrelationships between poor sleep, genetic susceptibility, and dementia merit further exploration.

Our study targeted rural-dwelling Chinese older adults, to whom insufficient attention has been paid so far by the research community, and we performed comprehensive assessments of self-reported sleep characteristics and cognitive function. Our study also has limitations. First, the cross-sectional design prevents us from making any causal inferences regarding the associations between self-reported sleep characteristics and cognitive outcomes, and the observed cross-sectional associations may be subject to selective survival bias. Instead, our study aimed to characterize the self-reported sleep characteristics associated with dementia among rural-dwelling Chinese older adults. Second, multiple testing could increase the possibility of detecting the false positive associations, although the main analyses were driven primarily by our research hypothesis. Third, sleep characteristics were assessed retrospectively through self-report, which might be subject to recall bias, especially for people with dementia, although PSQI and ESS were recommended to assess sleep characteristics among individuals with dementia via self-report [32]. Of note, the majority (~ 93%) of dementia cases in our study were classified to have mild dementia (i.e., Clinical Dementia Rating Scale score ≤ 1). In this regard, findings from our study might not be applicable to all patients with dementia from the general rural population owing to this selection bias. In addition, we also found associations between abnormal sleep characteristics and worse cognitive performance among dementia-free participants, which partly supported the observed associations between certain sleep characteristics and dementia. Nevertheless, given that participants in our analytical sample were relatively younger and received more years of education than the target population, our results might have been affected by selection bias, which might lead to overestimations of the association between sleep problems and dementia. Fourth, due to the relatively high proportion of illiterate persons in our study participants, some dementia cases might have been misclassified. However, our comprehensive diagnostic procedure for dementia (e.g., twice face-to-face interviews) might help minimize the misclassification [38]. Finally, we did not have a standard approach to define sleep apnea, despite its strong association with dementia.

## Conclusions

Our study shows that certain self-reported sleep characteristics (e.g., very short and long sleep duration and low sleep efficiency) are independently associated with dementia and poor global cognitive performance in Chinese rural older adults, in which the associations may vary by age and *APOE* ε4 status. This suggests that sleep characteristics associated with dementia and poor global cognitive function among Chinese rural older adults were similar to those reported from urban elderly residents. Future long-term prospective studies are needed to clarify their temporal and causal relationships, in which self-reported sleep characteristics can be integrated with those from objective assessments (e.g., polysomnography and actigraphy).

## Supplementary Information

**Additional file 1: Supplementary Table 1.** Associations of self-reported sleep characteristics with all-cause dementia and Alzheimer’s disease stratified by age groups (*n* = 4742). **Supplementary Table 2.** Associations of self-reported sleep characteristics with all-cause dementia and Alzheimer’s disease stratified by sex (n = 4742). **Supplementary Table 3.** Associations of self-reported sleep characteristics with all-cause dementia and Alzheimer’s disease stratified by education (n = 4742). **Supplementary Table 4.** Associations of self-reported sleep characteristics with all-cause dementia and Alzheimer’s disease stratified by *APOE* genotype (*n* = 4599).

## Data Availability

Human genetic data from MIND-China are unsuitable for public deposition due to current regulations. However, the datasets used and/or analysed during the current study are available from the corresponding author upon reasonable request and approval by the MIND-China Steering Committee.

## Abbreviations

AD: Alzheimer’s disease
ADLs: Activities of daily living
*APOE*: Apolipoprotein E gene
ATC: Anatomical Therapeutic Chemical
Aβ: Amyloid β
BMI: Body mass index
CI: Confidence interval
DSM-IV: The Diagnostic and Statistical Manual of Mental Disorders, Fourth Edition
ECG: Electrocardiogram
EDS: Excessive daytime sleepiness
ESS: Epworth Sleepiness Scale
GDS-15: The 15-item Geriatric Depression Scale
HDL-C: High-density lipoprotein cholesterol
LDL-C: Low-density lipoprotein cholesterol
MIND-China: Multimodal Interventions to Delay Dementia and Disability in Rural China
MMSE: Mini-Mental State Examination
OR: Odds ratio
the MrOS Sleep Study: The Outcomes of Sleep Disorders in Older Men Sleep Study
PSQI: Pittsburgh Sleep Quality Index
Ref.: Reference
TC: Total cholesterol
TG: Triglycerides
