# Supplementary material for: Self-reported sleep characteristics associated with dementia among rural-dwelling Chinese older adults: a population-based study
Source: BMC Neurol. 2022 Jan 3;22:5. doi: 10.1186/s12883-021-02521-0 (PMC8722012; doi:10.1186/s12883-021-02521-0)
Supplement: Supplementary file 1 — Additional file 1: Supplementary Table 1. Associations of self-reported sleep characteristics with all-cause dementia and Alzheimer’s disease stratified by age groups (n = 4742). Supplementary Table 2. Associations of self-reported sleep characteristics with all-cause dementia and Alzheimer’s disease stratified by sex (n = 4742). Supplementary Table 3. Associations of self-reported sleep characteristics with all-cause dementia and Alzheimer’s disease stratified by education (n = 4742). Supplementary Table 4. Associations of self-reported sleep characteristics with all-cause dementia and Alzheimer’s disease stratified by APOE genotype (n = 4599). [file 12883_2021_2521_MOESM1_ESM.docx]

**Additional File: Supplementary Table 1.** Associations of self-reported sleep characteristics with all-cause dementia and Alzheimer’s disease stratified by age groups (n=4742).

**Additional File: Supplementary Table 2.** Associations of self-reported sleep characteristics with all-cause dementia and Alzheimer’s disease stratified by sex (n=4742).

**Additional File: Supplementary Table 3.** Associations of self-reported sleep characteristics with all-cause dementia and Alzheimer’s disease stratified by education (n=4742).

**Additional File: Supplementary Table 4.** Associations of self-reported sleep characteristics with all-cause dementia and Alzheimer’s disease stratified by *APOE* genotype (n=4599).

**Supplementary Table 1** Associations of self-reported sleep characteristics with all-cause dementia and Alzheimer’s disease stratified by age groups (n=4742)

| Self-reported sleep characteristics | All-cause dementia (n=173) | | |  | Alzheimer’s disease (n=115) | | |
| --- | --- | --- | --- | --- | --- | --- | --- |
|  | Odds ratio (95% confidence interval)^a^ | | *p* for interaction |  | Odds ratio (95% confidence interval)^a^ | | *p* for interaction |
|  | <75 years | ≥75 years |  |  | <75 years | ≥75 years |  |
| Sleep duration |  |  | 0.166 |  |  |  | 0.298 |
| ≤4 h | 2.78 (1.54-5.02)^***^ | 0.79 (0.36-1.76) | 0.028 |  | 3.03 (1.46-6.32)^**^ | 0.97 (0.42-2.25) | 0.079 |
| >4 to 6 h | 0.82 (0.45-1.50) | 0.63 (0.32-1.25) | 0.758 |  | 0.97 (0.46-2.04) | 0.67 (0.31-1.44) | 0.665 |
| >6 to 8 h | 1.00 (Reference) | 1.00 (Reference) |  |  | 1.00 (Reference) | 1.00 (Reference) |  |
| >8 h | 1.76 (0.96-3.24) | 1.42 (0.72-2.81) | 0.556 |  | 1.98 (0.91-4.32) | 0.97 (0.41-2.26) | 0.222 |
| Sleep quality |  |  | 0.247 |  |  |  | 0.917 |
| Good | 1.00 (Reference) | 1.00 (Reference) |  |  | 1.00 (Reference) | 1.00 (Reference) |  |
| Poor | 1.46 (0.91-2.34) | 0.87 (0.50-1.51) |  |  | 1.30 (0.73-2.32) | 1.30 (0.68-2.48) |  |
| Sleep efficiency (per 10% decrease) | 1.22 (1.10-1.35)^***^ | 0.97 (0.85-1.10) | 0.020 |  | 1.21 (1.07-1.37)^**^ | 1.04 (0.90-1.21) | 0.190 |
| Sleep latency |  |  | 0.451 |  |  |  | 0.481 |
| ≤30 min | 1.00 (Reference) | 1.00 (Reference) |  |  | 1.00 (Reference) | 1.00 (Reference) |  |
| >30 min | 1.29 (0.82-2.04) | 1.00 (0.58-1.72) |  |  | 1.58 (0.90-2.77) | 1.32 (0.71-2.44) |  |
| EDS |  |  | 0.930 |  |  |  | 0.911 |
| No | 1.00 (Reference) | 1.00 (Reference) |  |  | 1.00 (Reference) | 1.00 (Reference) |  |
| Yes | 1.93 (1.13-3.29)^*^ | 1.86 (0.76-4.57) |  |  | 2.23 (1.15-4.35)^*^ | 2.06 (0.74-5.74) |  |

^a^Odds ratio and 95% confidence interval were derived from the models that were adjusted for age, sex, education, body mass index, alcohol consumption status, smoking status, leisure-time physical activity, hypertension, diabetes, dyslipidemia, coronary heart disease, stroke, depressive symptoms, hypnotics use, and *APOE* genotype.

^*^*p*<0.05, ^**^*p*<0.01, ^***^*p*<0.001.

*Abbreviations: EDS* excessive daytime sleepiness.**Supplementary Table 2** Associations of self-reported sleep characteristics with all-cause dementia and Alzheimer’s disease stratified by sex (n=4742)

| Self-reported sleep characteristics | All-cause dementia (n=173) | | |  | Alzheimer’s disease (n=115) | | |
| --- | --- | --- | --- | --- | --- | --- | --- |
|  | Odds ratio (95% confidence interval)^a^ | | *p* for interaction |  | Odds ratio (95% confidence interval)^a^ | | *p* for interaction |
|  | Men | Women |  |  | Men | Women |  |
| Sleep duration |  |  | 0.120 |  |  |  | 0.499 |
| ≤4 h | 2.67 (0.84-8.48) | 1.54 (0.91-2.59) | 0.426 |  | 3.08 (0.57-16.63) | 1.57 (0.88-2.80) | 0.432 |
| >4 to 6 h | 1.61 (0.71-3.64) | 0.61 (0.36-1.05) | 0.059 |  | 1.84 (0.58-5.87) | 0.72 (0.39-1.31) | 0.128 |
| >6 to 8 h | 1.00 (Reference) | 1.00 (Reference) |  |  | 1.00 (Reference) | 1.00 (Reference) |  |
| >8 h | 1.39 (0.56-3.45) | 1.84 (1.09-3.09)^*^ | 0.579 |  | 2.64 (0.78-8.89) | 1.29 (0.66-2.51) | 0.381 |
| Sleep quality |  |  | 0.036 |  |  |  | 0.468 |
| Good | 1.00 (Reference) | 1.00 (Reference) |  |  | 1.00 (Reference) | 1.00 (Reference) |  |
| Poor | 2.64 (1.29-5.40)^**^ | 0.92 (0.61-1.39) |  |  | 1.71 (0.66-4.41) | 1.19 (0.73-1.95) |  |
| Sleep efficiency (per 10% decrease) | 1.23 (1.02-1.47)^*^ | 1.09 (1.00-1.20)^*^ | 0.310 |  | 1.06 (0.81-1.39) | 1.14 (1.03-1.26)^*^ | 0.737 |
| Sleep latency |  |  | 0.190 |  |  |  | 0.496 |
| ≤30 min | 1.00 (Reference) | 1.00 (Reference) |  |  | 1.00 (Reference) | 1.00 (Reference) |  |
| >30 min | 1.96 (0.94-4.10) | 0.96 (0.65-1.44) |  |  | 1.64 (0.59-4.53) | 1.25 (0.79-1.98) |  |
| EDS |  |  | 0.842 |  |  |  | 0.209 |
| No | 1.00 (Reference) | 1.00 (Reference) |  |  | 1.00 (Reference) | 1.00 (Reference) |  |
| Yes | 1.61 (0.69-3.78) | 1.80 (1.04-3.13)^*^ |  |  | 0.91 (0.22-3.68) | 2.57 (1.40-4.70)^**^ |  |

^a^Odds ratio and 95% confidence interval were derived from the models that were adjusted for age, education, body mass index, alcohol consumption status, smoking status, leisure-time physical activity, hypertension, diabetes, dyslipidemia, coronary heart disease, stroke, depressive symptoms, hypnotics use, and *APOE* genotype.

^*^*p*<0.05, ^**^*p*<0.01, ^***^*p*<0.001.

*Abbreviations: EDS* excessive daytime sleepiness.

**Supplementary Table 3** Associations of self-reported sleep characteristics with all-cause dementia and Alzheimer’s disease stratified by education (n=4742)

| Self-reported sleep characteristics | All-cause dementia (n=173) | | |  | Alzheimer’s disease (n=115) | | |
| --- | --- | --- | --- | --- | --- | --- | --- |
|  | Odds ratio (95% confidence interval)^a^ | | *p* for interaction |  | Odds ratio (95% confidence interval)^a^ | | *p* for interaction |
|  | Illiteracy | Non-illiteracy |  |  | Illiteracy | Non-illiteracy |  |
| Sleep duration |  |  | 0.589 |  |  |  | 0.339 |
| ≤4 h | 1.60 (0.92-2.78) | 1.68 (0.68-4.17) | 0.808 |  | 1.70 (0.92-3.12) | 1.61 (0.45-5.76) | 0.984 |
| >4 to 6 h | 0.68 (0.39-1.18) | 0.91 (0.42-1.95) | 0.487 |  | 0.74 (0.40-1.37) | 1.06 (0.37-3.02) | 0.548 |
| >6 to 8 h | 1.00 (Reference) | 1.00 (Reference) |  |  | 1.00 (Reference) | 1.00 (Reference) |  |
| >8 h | 1.30 (0.74-2.29) | 2.38 (1.10-5.14)^*^ | 0.181 |  | 1.01 (0.50-2.03) | 2.83 (1.00-7.96)^*^ | 0.089 |
| Sleep quality |  |  | 0.691 |  |  |  | 0.378 |
| Good | 1.00 (Reference) | 1.00 (Reference) |  |  | 1.00 (Reference) | 1.00 (Reference) |  |
| Poor | 1.26 (0.81-1.95) | 1.08 (0.57-2.05) |  |  | 1.43 (0.86-2.40) | 1.02 (0.44-2.34) |  |
| Sleep efficiency (per 10% decrease) | 1.12 (1.02-1.23)^*^ | 1.07 (0.91-1.26) | 0.521 |  | 1.15 (1.03-1.28)^*^ | 1.03 (0.83-1.28) | 0.313 |
| Sleep latency |  |  | 0.329 |  |  |  | 0.581 |
| ≤30 min | 1.00 (Reference) | 1.00 (Reference) |  |  | 1.00 (Reference) | 1.00 (Reference) |  |
| >30 min | 1.26 (0.83-1.93) | 0.93 (0.48-1.80) |  |  | 1.44 (0.89-2.33) | 1.36 (0.57-3.24) |  |
| EDS |  |  | 0.886 |  |  |  | 0.371 |
| No | 1.00 (Reference) | 1.00 (Reference) |  |  | 1.00 (Reference) | 1.00 (Reference) |  |
| Yes | 2.09 (1.18-3.70)^*^ | 1.47 (0.68-3.16) |  |  | 2.48 (1.32-4.65)^**^ | 1.16 (0.36-3.71) |  |

^a^Odds ratio and 95% confidence interval were derived from the models that were adjusted for age, sex, education (years), body mass index, alcohol consumption status, smoking status, leisure-time physical activity, hypertension, diabetes, dyslipidemia, coronary heart disease, stroke, depressive symptoms, hypnotics use, and *APOE* genotype.

^*^*p*<0.05, ^**^*p*<0.01, ^***^*p*<0.001.

*Abbreviations: EDS* excessive daytime sleepiness.

**Supplementary Table 4** Associations of self-reported sleep characteristics with all-cause dementia and Alzheimer’s disease stratified by *APOE* genotype (n=4599)

| Self-reported sleep characteristics | All-cause dementia (n=172) | | |  | Alzheimer’s disease (n=114) | | |
| --- | --- | --- | --- | --- | --- | --- | --- |
|  | Odds ratio (95% confidence interval)^a^ | | *p* for interaction |  | Odds ratio (95% confidence interval)^a^ | | *p* for interaction |
|  | *APOE* ε4 allele non-carriers | *APOE* ε4 allele carriers |  |  | *APOE* ε4 allele non-carriers | *APOE* ε4 allele carriers |  |
| Sleep duration |  |  | 0.055 |  |  |  | 0.033 |
| ≤4 h | 1.31 (0.76-2.25) | 4.69 (1.41-15.57)^*^ | 0.023 |  | 1.23 (0.64-2.33) | 6.76 (1.51-30.33)^*^ | 0.028 |
| >4 to 6 h | 0.78 (0.48-1.26) | 0.60 (0.16-2.29) | 0.992 |  | 0.92 (0.53-1.60) | 0.53 (0.08-3.74) | 0.422 |
| >6 to 8 h | 1.00 (Reference) | 1.00 (Reference) |  |  | 1.00 (Reference) | 1.00 (Reference) |  |
| >8 h | 1.36 (0.83-2.23) | 4.01 (1.17-13.72)^*^ | 0.137 |  | 1.19 (0.64-2.21) | 3.73 (0.72-19.39) | 0.355 |
| Sleep quality |  |  | 0.098 |  |  |  | 0.046 |
| Good | 1.00 (Reference) | 1.00 (Reference) |  |  | 1.00 (Reference) | 1.00 (Reference) |  |
| Poor | 1.06 (0.71-1.57) | 1.80 (0.73-4.41) |  |  | 1.06 (0.66-1.71) | 4.92 (1.21-19.94)^*^ |  |
| Sleep efficiency (per 10% decrease) | 1.08 (0.99-1.18) | 1.22 (1.02-1.47)^*^ | 0.051 |  | 1.08 (0.97-1.20) | 1.32 (1.04-1.67)^*^ | 0.057 |
| Sleep latency |  |  | 0.014 |  |  |  | <0.001 |
| ≤30 min | 1.00 (Reference) | 1.00 (Reference) |  |  | 1.00 (Reference) | 1.00 (Reference) |  |
| >30 min | 0.97 (0.65-1.44) | 2.62 (1.08-6.38)^*^ |  |  | 1.00 (0.63-1.59) | 12.01 (2.78-51.96)^***^ |  |
| EDS |  |  | -^b^ |  |  |  | -^b^ |
| No | 1.00 (Reference) | 1.00 (Reference) |  |  | 1.00 (Reference) | 1.00 (Reference) |  |
| Yes | 2.47 (1.55-3.93)^***^ | -^b^ |  |  | 2.67 (1.53-4.69)^***^ | -^b^ |  |

Participants who had missing data on *APOE* genotype were excluded from the *APOE* genotype-stratified analyses (n=143).

^a^Odds ratio and 95% confidence interval were derived from the models that were adjusted for age, sex, education, body mass index, alcohol consumption status, smoking status, leisure-time physical activity, hypertension, diabetes, dyslipidemia, coronary heart disease, stroke, depressive symptoms, and hypnotics use. ^b^Not applicable or there was no case of dementia or Alzheimer’s disease.

^*^*p*<0.05, ^**^*p*<0.01, ^***^*p*<0.001.

*Abbreviations: EDS* excessive daytime sleepiness.
